# Antiviral activity of an *N*-allyl acridone against dengue virus

**DOI:** 10.1186/s12929-015-0134-2

**Published:** 2015-04-17

**Authors:** María B Mazzucco, Laura B Talarico, Sezen Vatansever, Ana C Carro, Mirta L Fascio, Norma B D’Accorso, Cybele C García, Elsa B Damonte

**Affiliations:** Laboratorio de Virología, Departamento de Química Biológica, Facultad de Ciencias Exactas y Naturales, UBA, Ciudad Universitaria, Piso 4, Buenos Aires, Pabellón 1428 Argentina; Graduate School of Science and Engineering, Koc University, Rumelifener yolu, Istanbul, Sarıyer 34450 Turke; IQUIBICEN-Consejo Nacional de Investigaciones Científicas y Técnicas (CONICET), Buenos Aires, Argentina; CIHIDECAR (CONICET), Departamento de Química Orgánica, Facultad de Ciencias Exactas y Naturales, UBA, Ciudad Universitaria, Pabellón 2, Piso 3, Buenos Aires, 1428 Argentina; Present address: Laboratorio de Reproducción y Metabolismo, Facultad de Medicina, CEFYBO-CONICET, UBA, Buenos Aires, 1121 Argentina; Present address: Fundación Infant, Buenos Aires, 1406 Argentina

**Keywords:** Dengue virus, Antiviral, Re-emerging infection, Acridone, RNA synthesis

## Abstract

**Background:**

Dengue virus (DENV), a member of the family *Flaviviridae*, is at present the most widespread causative agent of a human viral disease transmitted by mosquitoes. Despite the increasing incidence of this pathogen, there are no antiviral drugs or vaccines currently available for treatment or prevention. In a previous screening assay, we identified a group of *N*-allyl acridones as effective virus inhibitors. Here, the antiviral activity and mode of action targeted to viral RNA replication of one of the most active DENV-2 inhibitors was further characterized.

**Results:**

The compound 10-allyl-7-chloro-9(10*H*)-acridone, designated **3b**, was active to inhibit the *in vitro* infection of Vero cells with the four DENV serotypes, with effective concentration 50% (EC_50_) values in the range 12.5-27.1 μM, as determined by virus yield inhibition assays. The compound was also effective in human HeLa cells. No cytotoxicity was detected at **3b** concentrations up to 1000 μM. Mechanistic studies demonstrated that virus entry into the host cell was not affected, whereas viral RNA synthesis was strongly inhibited, as quantified by real time RT-PCR. The addition of exogenous guanosine together with **3b** rescued only partially the infectivity of DENV-2.

**Conclusions:**

The acridone derivative **3b** selectively inhibits the infection of Vero cells with the four DENV serotypes without a direct interaction with the host cell or the virion but interfering specifically with the intracellular virus multiplication. The mode of antiviral action for this acridone apparently involves the cellular enzyme inosine-monophospahe dehydrogenase together with another still unidentified target related to DENV RNA synthesis.

## Background

The four serotypes of dengue virus (DENV-1 - DENV-4) are the most important mosquito-borne human pathogens included in the family *Flaviviridae*. The World Health Organization estimates that DENV infects annually 50 million people around the world causing a wide range of clinical disease, from the benign and autolimited dengue fever to the severe forms of dengue hemorrhagic fever and dengue shock syndrome [1,2]. However, new appraisals indicate that apparent and inapparent DENV infections would reach the number of 350 million per year [3,4]. The virion contains a positive-sense single stranded RNA inserted in an icosahedral nucleocapsid and surrounded by a lipid envelope. The genome is translated into three structural proteins (the envelope E glycoprotein, the membrane M and the capsid C proteins) and seven non-structural proteins (NS1, NS2A, NS2B, NS3, NS4A, NS4B and NS5).

No specific anti-DENV drugs or vaccines are currently available. The present treatment for DENV infected patients consists only in supportive medical care directed to reduce the symptoms and to improve survival in the severe forms of disease. Given the worldwide expansion of DENV reemergence in different geographical regions and the consequent increase in at risk population, there is an urgent need of effective compounds to control morbidity and mortality caused by this flavivirus. To date, several strategies have been intended for development of anti-DENV agents, directed to block either a virus encoded protein or a host cell factor required for virus multiplication and/or pathogenesis [5,6]. Although the estimated global prevalence of dengue is very high, very few clinical trials have been performed. The candidate drugs just tested in humans include the lysosomotropic agent chloroquine [7] and the polymerase inhibitor balapiravir [8], both without therapeutic efficacy, whereas trials of lovastatin, a cholesterol-reducing agent, and cellular glucosidase inhibitors are currently underway [9,10].

As occurs with other RNA viruses, the inhibition of intracellular DENV RNA synthesis has been one of the most explored approaches for antiviral studies. The proteins NS3 and NS5 are engaged in viral RNA replication: the NS5 conserved protein has a methyltransferase activity in the N-terminal domain, involved in RNA cap formation, and an RNA-dependent RNA polymerase in the C-terminal domain [11,12], whereas the C-terminal portion of NS3 comprises 5’RNA triphosphatase, RNA helicase and nucleoside triphosphatase activities [13]. Both NS3 and NS5 have been structurally characterized and are possible antiviral targets [12,14,15]. Furthermore, diverse compounds interacting with host cell components participating in viral RNA replication or transcription, like cellular proteins interacting with viral RNA or involved in the *de novo* biosynthesis of purine or pyrimidine nucleotides, have also been investigated [16].

**Figure 1:**
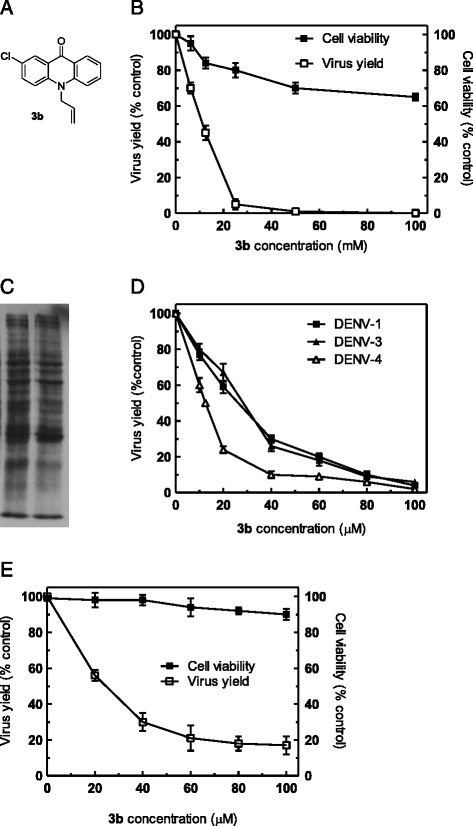
Cytotoxicity and antiviral activity of **3b**. **(A)** Chemical structure of compound **3b**. **(B)** Vero cells were incubated for 96 h in the presence of different concentrations of **3b** and then cell viability was determined by MTT assay (■). Other set of cultures were infected with DENV-2 (MOI 0.1 PFU/cell) in the presence or absence of **3b** and virus yields were determined at 48 h p.i. (□). **(C)** Effect of **3b** on cell protein synthesis. Vero cells were incubated for 48 h with or without 100 μM **3b**. Then, cells were labeled with 100 μCi/ml EXPRE^35^S-^35^S for 4 h and polypeptides were electrophoresed. **(D)** Vero cells were infected with DENV-1 (■), DENV-3(▲) or DENV-4 (∆) at a MOI of 0.1 PFU/cell in the presence or absence of **3b** and virus yields were determined at 48 h p.i. **(E)** Cytotoxicity (■) and antiviral activity (□) against DENV-2 (MOI 0.1 PFU/cell) were determined in HeLa cells as in (B). In (B), (D) and (E) results are expressed as % cell viability or virus yield with respect to control without drug treatment and represent the mean of three independent experiments ± standard deviation.

Regarding viral RNA inhibition, acridones are a class of heterocyclic compounds that have attracted attention in recent years for their wide range of biological properties centered on the synthesis of nucleic acids, including anti-cancer properties and inhibitory action against DNA and RNA viruses [17-20]. In a previous screening assay of antiviral activity against DENV-2 of a series of *N*-substituted acridones, we identified a group of *N*-allyl derivatives as effective virus inhibitors [21]. Here, the antiviral activity and mode of action targeted to viral RNA replication of one of the most active DENV-2 inhibitors, the 10-allyl-7-chloro-9(10*H*)-acridone designated **3b** (Figure 1A), was further characterized.

## Methods

### Compounds

The acridone derivative **3b** was synthesized as previously described [21]. Stock solution at a concentration of 100 mM was prepared in dimethylsulfoxide. Ribavirin (RIB) was purchased at Sigma-Aldrich and was also dissolved in dimethylsulfoxide.

### Cells and viruses

Vero (African green monkey kidney) and HeLa (human cervical carcinoma) cells were grown in Eagle’s minimum essential medium (MEM) (GIBCO) supplemented with 5% fetal bovine serum. For maintenance medium, the serum concentration was reduced to 1.5%. The C6/36 mosquito cell line from *Aedes albopictus,* adapted to grow at 33°C, was cultured in L-15 medium (Leibovitz) supplemented with 0.3% tryptose phosphate broth, 0.02% glutamine, 1% MEM non-essential amino acids solution and 10% fetal bovine serum.

Virus stocks of DENV-1 strain Hawaii, DENV-2 strain New Guinea C (NGC), DENV-3 strain H87 and DENV-4 strain 8124 were prepared in C6/36 cells and titrated by plaque formation in Vero cells.

### Antiviral assay

Antiviral activity was determined by a virus yield inhibition assay. Vero cells grown in 24-well plates were infected at a multiplicity of infection (MOI) of 0.1 PFU/cell. After 1 h at 37°C, cells were washed and refed with maintenance medium containing or not serial two-fold dilutions of compound. After 48 h of incubation at 37°C, extracellular DENV yields were determined by plaque forming units (PFU) titration in Vero cells. In a similar assay, we have previously determined that treatment with medium containing dimethylsulfoxide 1:100–1:10000 (solvent dilutions corresponding to the working solutions of compound) did not affect DENV infectivity. The effective concentration 50% (EC_50_) was calculated as the concentration required to reduce virus yield by 50% in the compound-treated cultures compared with untreated ones using a non linear regression of dose response inhibition with GraphPad Prism software. All determinations were performed thrice, and each time in duplicate.

### Cell viability assay

Vero and HeLa cell cultures grown in 96-well plates (5 × 10^4^ cells/well) were exposed for 96 h to serial two-fold compound dilutions, three wells for each concentration. Then, viability was measured by the 3-(4,5-dimethylthiazol-2-yl)-2,5-diphenyl tetrazolium bromide (MTT, Sigma-Aldrich) method as previously described [22]. The cytotoxic concentration 50% (CC_50_) is the compound concentration required to reduce the MTT signal by 50% compared to untreated controls, calculated as mentioned for EC_50_. All determinations were performed thrice and each one in duplicate.

### Cell protein synthesis

Vero cells were incubated in the presence or absence of 100 μM **3b** during 48 h at 37°C. Then, cells were washed with phosphate buffer and incubated in methionine-cysteine-free medium in the presence or absence of compound for 1 h, and then labelled by addition of 100 μCi/ml of EXPRE^35^S-^35^S (NEN Dupont) for 4 h. After labelling, cells were lysed in sample electrophoresis buffer (5% sodium dodecyl sulfate, 2% 2-mercaptoethanol, 10% glycerol, and 0.005% bromophenol blue in 0.0625 M Tris–HCl, pH 6.8). Cell lysates were sonicated for 1 min, boiled during 2 min and loaded for electrophoresis on 15% SDS-polyacrylamide gels (lysates corresponding to 1.0 × 10^5^ cells/well). Protein bands were visualized by fluorography.

### Effect of treatment of cells or virus with 3b before infection

Pretreatment of cells: Vero cells were pre-incubated with maintenance medium containing or not 100 μM **3b** for 2 h at 37°C. Then, cells were thoroughly washed with medium and infected with DENV-2 (MOI of 0.1) in the absence of compound. Virus yields were determined at 48 h p.i. by PFU. Pretreatment of virus: a DENV-2 suspension (1 × 10^6^ PFU/ml) was incubated in MEM containing or not 100 μM **3b** for 1.5 h at 37°C. Then, samples were diluted and the remaining infectivity was titrated by PFU. Treatment during virus infection: Vero cells were infected with DENV-2 with DENV-2 and after adsorption medium containing **3b** was added and maintained during 48 h at 37°C, when virus yields were determined.

### Virus adsorption and internalization

For evaluation of virus adsorption, Vero cells grown in coverslips were infected with DENV-2 (MOI 1 PFU/cell) in the presence or absence of 100 μM **3b**. After 15 min adsorption at 4°C, cell monolayers were washed with cold phosphate buffer and fixed in methanol for 15 min at −20°C for cytoplasmic immunofluorescence. Indirect staining was carried out by using mouse monoclonal antibody against DENV E glycoprotein (Abcam) and fluorescein isothiocyanate-labeled goat anti-mouse IgG (Sigma-Aldrich). After a final washing with phosphate buffer, cells were stained with Evans Blue and mounted in a glycerol solution containing 1,4-diazabicyclo [2] octane.

For virus internalization assay, Vero cells grown in coverslips were infected with DENV-2 (MOI 1 PFU/cell). After 1 h adsorption at 4°C, cells were washed with cold phosphate buffer and incubated at 37°C for 1 h in medium containing or not 100 μM **3b**. Then, cells were fixed in methanol and internalized viral protein was stained for indirect immunofluorescence as above.

### Expression of viral proteins

Vero cells grown in coverslips were infected with DENV-2 (MOI 1 PFU/cell) in the presence or absence of 100 μM **3b**. At 48 h p.i., cells were fixed and processed for cytoplasmic immunofluorescence as above described.

### Synthesis of viral RNA

Vero cells were infected with DENV-2 at a MOI of 1 PFU/cell and after adsorption cells were refed with maintenance medium containing or not 100 μM **3b**. At 8, 24 and 48 h p.i., total RNA was extracted by using TRIZOL (Invitrogen Life Technologies) according to the manufacturer’s instructions. Then, cDNA was generated by using murine reverse transcriptase M-MLV (100 U/μl, Invitrogen) and random primers. This cDNA was amplified by real time PCR using SYBRGreen (Roche) detection. The mix reaction volume was 25 μl including 2 μl of cDNA, DNA polymerase GoTaq (5 U/μl, Promega) and specific primers to amplify the gene of NS1 protein (the sense primer was 5’CGATCTCTCAGCCCCAGCCCACTGAGC3’ and the antisense, 5’CCATGTGTCATTGAGTGCACTTTCTATCC3’). Real time PCR was carried out with an initial incubation at 95°C during 3 min, followed by 40 cycles of 15 s at 95°C, 1 min at 61°C and 30 s at 72°C and 10 min at 72°C. Amplification plots were expressed as CT values to be analyzed with Opticon Monitor 3.1 software to calculate the relative amounts of viral RNA. The cellular gene *actin* was used as standard for normalization.

### Reversal by guanosine

To address the ability of guanosine to reverse the inhibitory effect on virus infection, DENV-2 was adsorbed to Vero cells at a MOI of 0.1 PFU/cell for 1 h. Then, cultures were incubated with maintenance medium containing a fixed concentration of **3b** (100 μM) or ribavirin (RIB) (200 μM) and variable concentrations of guanosine (0–1000 μM). Other set of Vero cell cultures were infected with DENV-2 as above and incubated with medium containing only guanosine (0–1000 μM). For all treatments, extracellular virus yields were determined after 48 h of infection by PFU.

### Molecular docking studies

Crystal structures of the proteins were obtained from Protein Data Bank. In Discovery studio (DS) program, proteins were modified by removing hetatoms from the structures. Ligand structure was checked with MarvinSketch and its geometry optimised by DS. Protein Data Bank file formats of the proteins and mol2 or sd file formats of the ligands were uploaded to all currently used softwares.

The active sites of the enzymes were determined by combining literature data with virtual screening observations. DS and Pymol were drived as virtual screening tools.

Molegro Virtual Docker (MVD) and GOLD molecular docking softwares were used together in a comparative manner. The searching space was specified that includes the active site properly. All explicit hydrogens were added into the structures. MolDock Score were used in MVD docking setup with the following settings: for the local search, MolDock Simplex Evolution algorithm was applied with a maximum of 2000 iterations per search. Fifty independent docking runs were carried out. Positions of the hydrogens for any hydrogen donors (both in **3b** and in the enzyme) were optimised. The Ignore Similar Poses option was used to avoid reporting to similar poses. All poses returned from the runs were clustered according to the root-mensquare deviation (RMSD) criterion and similar poses were removed (keeping the best-scoring one). Different poses of the compounds were ranked by their rerank scores and the best docked conformation was selected. More negative score indicates higher binding affinity. In Gold docking setup, Gold Genetic Algorithm was used with the slow option. ChemPLP (Piecewise Linear Potential) method was chosen as the scoring function and ChemScore was the rescoring function. Search options of Genetic Algorithm were automatically configured by the programme. Resulting poses were ranked by their PLR fitness functions and Chemscore ∆G values. In GOLD higher fitness score means better docked pose.

### Statistical analyses

Statistical analyses were performed using GraphPad Prism software. Comparison of means was tested by one-way analysis of variance (ANOVA) with Dunnett’s posttest. Statistical significance was defined as p < 0.05. Three asterisks (***), P ≤ 0.001.

## Results

### Spectrum of anti-DENV activity

**Figure 2:**
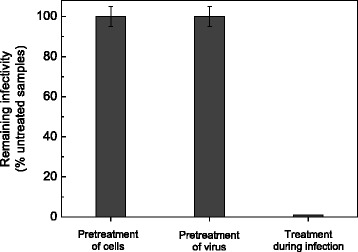
Effect of treatment of cells or virus with **3b** before and during infection. Pretreatment of cells: Vero cells were incubated with maintenance medium containing or not 100 μM **3b** for 2 h at 37°C, then compound was removed and cells were infected with DENV-2. Virus yields were determined at 48 h p.i. Pretreatment of virus: DENV-2 suspensions were incubated with MEM containing or not 100 μM **3b** for 1.5 h at 37°C and then remaining infectivity was determined by plaque assay. Treatment during infection: Compound was added to Vero cells after virus adsorption and maintained for 48 h at 37°C, when virus yields were determined. Results are expressed as percent of remaining infectivity in compound-treated samples compared to untreated ones. Each value is the mean of triplicate assays ± standard deviation.

The antiviral activity of **3b** against DENV-2 was evaluated by a virus yield reduction assay in Vero cells. As seen in Figure 2A, this compound exerted a dose-dependent inhibition of virus multiplication, attaining a reduction in DENV-2 yield relative to untreated infected cultures higher than 90% in the range of concentrations 50–100 μM.

By contrast, no important cytotoxic effects were detected by MTT method in Vero cell cultures after 96 h of incubation with the compound (Figure 1B). Furthermore, the cell treatment with **3b** up to a concentration of 1 mM, highly exceeding the effective antiviral concentration, almost did not alter Vero cell viability (data not shown).

The influence of **3b** on cell metabolism was further studied by analyzing cell protein synthesis after compound treatment. By radiolabeling of Vero cell cultures in the presence or absence of 100 μM **3b** and polypeptide detection by polyacrylamide-gel electrophoresis, only minimal alterations in intensity levels for certain protein bands were observed in acridone-treated cells in comparison with untreated ones (Figure 1C). These results are consistent with the very weak reduction in cell viability registered after treatment with 100 μM **3b** as shown by MTT assay in Figure 1B, confirming the lack of undesirable effects of **3b** on host cell and the selectivity of the observed anti-DENV activity.

It has been reported that the inhibitory effect against DENV of some compounds may be variable according to virus serotype [22-25]. Since all DENV serotypes co-circulate simultaneously in several tropical and subtropical areas, the antiviral activity against the four DENV serotypes was next evaluated by virus yield reduction assay. A dose-dependent inhibition similar to that presented for DENV-2 was obtained with the other serotypes (Figure 1D). The values of EC_50_ of **3b** for all serotypes, extrapolated from dose response curves, are presented in Table 1. Although slight variations are observed in antiviral susceptibility, the acridone exhibited a good degree of effectiveness against the four DENV serotypes, with selectivity indices (ratio between cytotoxicity and antiviral activity) in the range > 36.9 - > 80.0, values that warrant the potential perspectives of this type of compound as anti-DENV agent. These results confirm our previous report about screening of antiviral activity with diverse classes of substituted acridones that showed the inhibitory effect against DENV-1 to DENV-4 serotypes for other two different acridone derivatives [21].

**Table 1:** **Antiviral activity profile of 3b against DENV serotypes**

**DENV serotype**	**EC**_**50**_ **(μM)** ^**a**^	**SI** ^**b**^
DENV-1	26.7 ± 1.8	>37.3
DENV-2	13.5 ± 0.9	>74.1
DENV-3	27.1 ± 2.1	>36.9
DENV-4	12.5 ± 0.8	>80.0

^a^Effective concentration 50%: compound concentration required to reduce virus yields at 48 h p.i. by 50%. Values are the mean from triplicate independent tests ± standard deviation.

^b^Selectivity index: ratio CC_50_/EC_50_. The CC_50_ for **3b** is > 1000 μM.

The influence of the host cell on the antiviral inhibition was also analyzed. In particular, human-derived cells like the HeLa cell line was employed in the virus yield inhibition assay. The sensitivity of HeLa cells to the anti-DENV action of **3b** was comparable to that observed in Vero cells (Figure 1E). The EC_50_ against DENV-2 in HeLa cells was 23.9 ± 2.5 μM and, since the compound did not affect HeLa cell viability at concentrations up to 1000 μM as determined by MTT assay, the SI in HeLa cells was > 41.7, in the order of anti-DENV selectivity detected in Vero cells (Table 1).

### Acridone has no inhibitory action by cell or virus pretreatment

To characterize the mode of action of the acridone **3b** against DENV-2, we first investigated the possibility that the compound acted directly on the cells to be infected, inducing a cellular antiviral state, or directly on the virus, leading to inactivation of virion infectivity. The pre-incubation of Vero cells with **3b**, followed by compound removal and subsequent DENV-2 infection, did not produce any inhibition in virus yield at 48 h p.i. (Figure 2). Similarly, the acridone was not effective in reducing virus titer after 1 h of pre-incubation of a virus suspension with compound.

Only when the acridone was added to cells simultaneously with virus, DENV-2 yield was significantly reduced as seen in Figure 2. Then, **3b** failed either to induce a refractory state by cell pretreatment or to neutralize DENV-2, and the inhibitory effect was exclusively exerted through a blockade in virus multiplication during the course of infection.

### Effects of acridone on DENV-2 entry, RNA synthesis and protein expression

**Figure 3:**
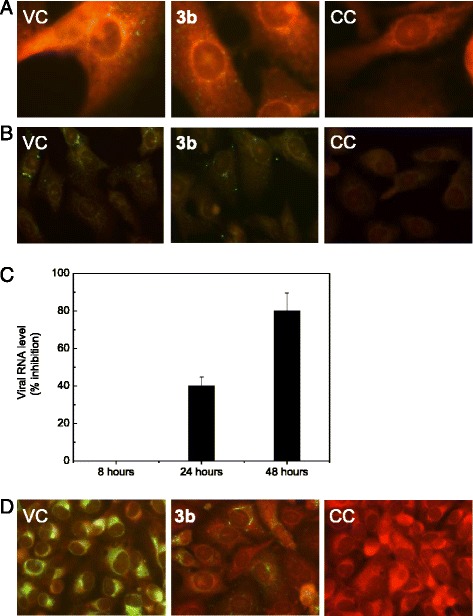
Effect of **3b** on DENV-2 entry, RNA synthesis and protein expression. **(A)** Vero cells were infected with DENV-2 in the absence or presence of 100 μM **3b**. After 15 min adsorption at 4°C indirect immunofluorescence staining of E glycoprotein was performed by using mouse monoclonal antibody against DENV E glycoprotein and fluorescein isothiocyanate-labeled goat anti-mouse IgG. Cells were stained with Evans blue. Magnification: 1000X. **(B)** DENV-2 was adsorbed to Vero cells for 1 h at 4°C adsorption. Then, cells were incubated at 37°C for 1 h in medium containing or not 100 μM **3b**, and thereafter cells were stained for indirect immunofluorescence as above. Magnification: 400X. **(C)** Vero cells were infected with DENV-2 and incubated for 8, 24 and 48 h in the absence or presence of 100 μM **3b**. At each time point, total RNA was extracted and cDNA was synthesized with random primers. These cDNAs were amplified by real time PCR using specific primers to amplify the *ns1* gene, and cellular *actin* was used for normalization. Results are expressed as % inhibition DENV-2 RNA level respect to viral control. **(D)** Vero cells were infected with DENV-2 in the absence (virus control VC) or presence (**3b**) of 100 μM **3b**. CC: cell control. At 48 h p.i. indirect immunofluorescence staining of E glycoprotein was performed as in **(A)**. Magnification: 400X.

To understand the mechanism underlying acridone inhibition of DENV infection, we investigated the effect of **3b** on different steps of the virus multiplication cycle. The potent inhibition observed in experiments presented in Figures 1 and 2 was always obtained in the absence of compound during virus adsorption. The lack of effect of **3b** on DENV-2 adsorption was confirmed by immunofluorescent staining of bound viral proteins after 1 h of incubation at 4°C (Figure 3A). Next, the action of **3b** on DENV-2 internalization was studied. DENV-2 was adsorbed to Vero cells for 1 h at 4°C, then the compound was added and temperature of incubation was immediately raised to 37°C to allow virus internalization. Under these treatment conditions, the internalized DENV-2 proteins after 1 h at 37°C detected by immunofluorescence staining was similar in compound-treated and –untreated cells (Figure 3B).

Afterwards, viral macromolecular synthesis was evaluated. Real time PCR was employed to measure intracellular viral RNA at 8, 24 and 48 h p.i. in DENV-2 infected Vero cells in the presence or absence of 100 μM **3b**. In the first 8 h p.i., no significant difference in viral RNA profile was detected between **3b**-treated and untreated cells (Figure 3C), supporting that the compound did not block the initial events of DENV multiplication cycle. By contrast, data shown in Figure 3C show that acridone inhibited viral RNA synthesis by 40% at 24 h p.i. and about 80% inhibition in DENV-2 RNA accumulation was displayed at 48 h p.i. The differences observed in RNA inhibitory effect between early and later times may be indicative of a very low amount of viral RNA at 8 h p.i. that is not enough to visualize an inhibition in the presence of the drug. When incubation time is prolonged to 24–48 h, the amount of viral RNA in the cells allows the detection of a significative reduction in the presence of **3b**.

The failure on RNA synthesis was also corroborated by analyzing the effect of **3b** on the expression of DENV-2 proteins, determined by indirect immunofluorescence staining of E viral glycoprotein. A correlation between the effects of **3b** on DENV-2 RNA accumulation and protein expression in infected cells was observed with a drastic reduction at 48 h p.i. in the number of cells expressing DENV-2 protein as well as in the intensitity of cytoplasmic immunofluorescence in the few positive cells (Figure 3D).

### Reversal by guanosine on DENV infectivity

Acridone-based compounds have been reported to be potent and uncompetitive inosine monophosphate dehydrogenase (IMPDH) inhibitors [26]. IMPDH is a key enzyme responsible for catalyzing the conversion of inosine 5’-monophosphate to xanthosine 5’-monophosphate, a rate-limiting step for intracellular *de novo* synthesis of guanosine nucleotides.

**Figure 4:**
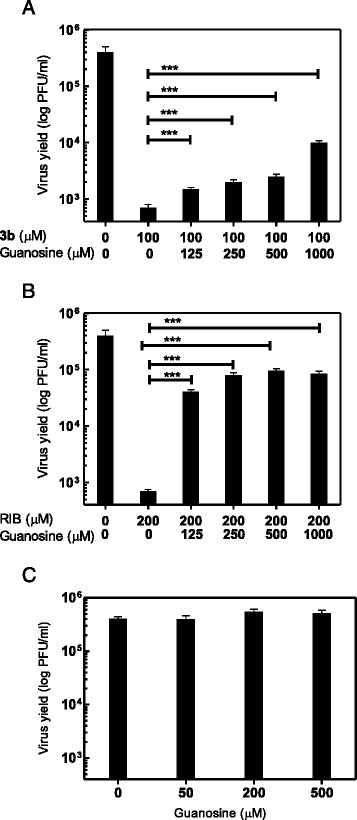
Reversal of inhibition by exogenous guanosine. Vero cells were infected with DENV-2 and treated with 100 μM **3b** **(A)** or 200 μM RIB **(B)** in the presence or absence of different guanosine concentrations. Other set of cultures were infected with DENV-2 and incubated with different concentrations of guanosine alone **(C)**. After 48 h, extracellular virus yields were determined by plaque formation. Each value is the mean of triplicate assays ± standard deviation. (***), P ≤ 0.001.

To explore whether the antiviral activity of **3b** might be due to GTP pool reduction, DENV-2 infected Vero cells were incubated with maintenance medium containing **3b,** in the presence or absence of exogenous guanosine. As control RIB, a compound with reported inhibitory activity against IMPDH [27,28], was simultaneously tested against DENV infection in the same conditions. The reversal assay was performed with a fixed concentration of each inhibitor that reduced virus titer by almost 3 log (Figure 4), and increasing concentrations of guanosine. The addition of exogenous guanosine rescued the infectivity of DENV in **3b**-treated cells in a dose-dependent manner, but the reversal was partial as reflected in PFU levels: virus yields were reduced from 2.85 × 10^4^ PFU/ml in the control infected cells to 7.0 × 10^2^ PFU/ml in **3b**-treated cells, and the simultaneous treatment with **3b** and 1000 μM guanosine increased the titer to 1.1 × 10^4^ PFU/ml. Thus, 1.16 log of infectivity was recovered but there was still a 1.44 log of viral inhibition in comparison to untreated infected cells (Figure 4A). On the other hand, the guanosine reversal for RIB was more complete with 2 log of infectivity recovered, and only 0.7 log of remaining viral yield inhibition (Figure 4B). To discard any benefitial effect of guanosine on DENV-2 replication and assess that the increased virus yields shown in Figure 4A and B are due to reversal of inhibition, Vero cells infected with DENV-2 were also incubated with different concentrations of guanosine in the absence of **3b** or RIB. As shown in Figure 4C, the treatment of infected cells with guanosine alone did not alter virus yield.

### Docking studies

**Figure 5:**
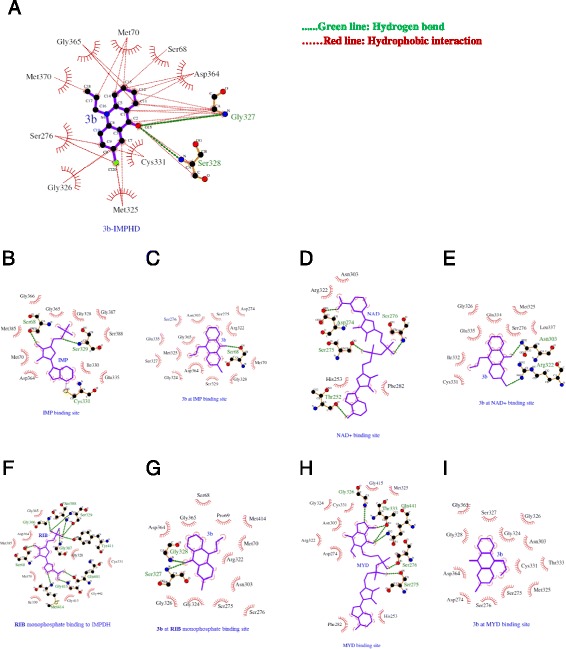
Docking modeling between the compound **3b** and the postulated target protein IMPDH. **(A) 3b** binds to the active site of the IMPHD enzyme through 2 hydrogen bonds and hydrophobic interaction. **(B)** IMP interaction cavity consists of SER68, MET70, GLY328, SER329, ILE330, CYS331, GLU335, ASP364, GLY365, GLY366, GLY387, SER388. **(C) 3b** made a hydrogen bond with SER68 and hydrophobic interactions with SER68, MET70, ASP274, SER275, SER276, ASN303, ARG322, GLY324, MET325, SER327, GLY328, SER329, GLU335, ASP364, GLY365. **(D)** NAD interaction pocket residues are THR45, THR252, HIS253, ASP274, SER275, SER276, PHE282, ASN303, ARG322, HIS466, GLN469. **(E)** Two hydrogen bonds existed between **3b** and ASN303, ARG322. **3b** also made hydrophobic interactions with SER276, ASN303, ARG322, MET325, GLY326, CYS331, ILE332, GLN334, LEU337. **(F)** RIB binding site includes SER68, MET70, ARG322, GLY328, SER329, ILE330, CYS331, ASP364, GLY365, GLY366, GLY387, SER388, TYR411, GLY413, MET414, GLY415, GLN441. **(G) 3b** made hydrogen bonds with SER327, GLY328 and hydrophobic interactions with PRO69, MET70, SER275, ASN303, ARG322, GLY324, GLY326, SER327, GLY328, ASP364, GLY365.**(H)** HIS253, ASP274, SER275, SER276, PHE282, ASN303, ARG322, GLY324, MET325, GLY326, CYS331, THR333 involves in MYD activity cavity. **(I) 3b** made hydrophobic interactions with ASP274, SER275, SER276, ASN303, GLY324, MET325, GLY326, SER327, GLY328, ASP364.

In order to explore if an interaction is possible at the molecular level, an extensive docking modeling was performed between the compound **3b** and the postulated target protein IMPDH. The 3D structure of human IMPHD was obtained from Protein Data Bank (Accession Number: 1NF7). Molegro and GOLD molecular docking softwares were used together for effective pose prediction and virtual screening. For determining the best pose, Molegro’s rerank scoring function and GOLD’s ChemPLP fitness score were used. Opposite of the rerank scoring, higher ChemPLP fitness score means better docked pose. Comparative docking results showed that the compound **3b** occupied the active site of IMPHD with a rerank score of −80.6446 and ChemPLP score of 51.0029, Chemscore ∆G of −25.0379. Two hydrogen bond interactions were identified with the backbone amino acid residues SER327 and GLY328. Moreover, 26 hydrophobic interactions with 11 residues including active site - CYS331- were observed (Figure 5A).

Next we analyzed how **3b** binds to native ligand’s interaction sites within the enzyme by using Molegro. Nicotinamide adenine dinucleotide (NAD) and inosine monophosphate (IMP) are chemically active native ligands of IMPDH which is an enzyme that catalyzes the NAD-dependent oxidation of IMP [29,30]. Redocking was applied to NAD and IMP for fair and accurate comparison. Complex form of IMPHD bound to its native ligands is stored in Protein Data Bank (Accession Number: 1NFB). After determining the residues which interact with NAD and IMP separately, ligands were removed from the 1NFB structure. Then NAD and IMP were docked back into the protein. **3b**-IMPHD docking was done by targeting the interaction regions of natural substrates.

It is known from the crystallographic studies that NAD binding site includes 11 structural amino acids (Risal D, Strickler MD, Goldstein BM, Crystal structure of human inosine monophosphate dehydrogenase type II complexed with the MPA/NAD analog C2-MAD, in preparation) (Figure 5B). Redocking of NAD gave −117.426 rerank score, conversely, docking of **3b** to this NAD binding site gave −67.4364 rerank score. Relatively low binding score of **3b** revealed that this site does not fit for it (Figure 5C). As for IMP, the substrate binds to the enzyme through CYS331 and the other 11 amino acids (Figure 5D). IMP’s docking back into IMPHD gave score of −112.119. When the compound **3b** was docked in the IMP activity region, it made a hydrogen bond with the side chain residue SER68 by resulting rerank score of −82.2138 (Figure 5E). Then, the results implied that **3b** showed higher binding affinity to IMP activity site than NAD activity site.

In addition to **3b**-NAD/IMP activity site interaction analysis, we investigated **3b** interactions with the inhibitory regions of two IMPDH antagonists: RIB monophosphate and C2-Mycophenolic Adenine Dinucleotide (MYD). RIB monophosphate and MYD show their antagonistic activity by blocking access to active site of IMPHD. Following the previous part’s method, the position of **3b** when it interacts with each binding region of those two inhibitors was figured out. The 3D structure of IMPHD- RIB monophosphate, MYD complex was obtained from Protein Data Bank (Accession number: 1NF7). As presented in the previous crystallographic study, RIB monophosphate and MYD bind to their inhibitory pockets by making hydrogen bonds and hydrophobic interactions (Risal D, Strickler MD, Goldstein BM, The conformation of NAD bound to human inosine monophosphate dehydrogenase type II, in preparation) (Figure 5 F,H). At first, they were docked back into their pockets. Best-fitting poses’ rerank scores were −116.695 for RIB monophosphate and −130.851for MYD. Later, **3b** was docked into each drug’s inhibitory pocket. **3b** presented high affinity to RIB monophosphate binding region by resulting rerank score of 82.2913. (Figure 5G). In contrast, it showed low affinity to MYD inhibitory cavity (Figure 5I).

## Discussion and conclusions

A preliminary structure-activity analysis with diverse substituted acridones showed that certain *N*-allyl acridone derivatives exerted antiviral activity against the hemorrhagic fever causing viruses DENV and Junin virus (JUNV), agent of Argentine hemorrhagic fever [21]. In particular, the halogen substituents in the acridone heterocyclic ring were found associated to reduced cytotoxicity and increased antiviral effects. Two 6-chloro derivatives, the 10-allyl-6-chloro-9(10*H*)-acridone and 10-allyl-6-chloro-4-methoxy-9(10*H*)-acridone, designated **3c** and **3f**, respectively, and one 7-chloro derivative, the 10-allyl-7-chloro-9(10*H*)-acridone or **3b**, were potent inhibitors of DENV-2 and JUNV infection at noncytotoxic concentrations [21]. In the present study, the anti-DENV activity of **3b** was pursued and the inhibition of the *in vitro* DENV infection without a direct interaction with the host cell or the virion was demonstrated. Furthermore, the antiviral effectiveness of **3b** appeared to be ascribed to an interference with viral RNA synthesis.

Natural and synthetic acridone-based substances have been known as multi-targeted agents with a wide spectrum of biomedical potential [17,19]. In particular, different acridone derivatives have been recently studied as potential inhibitors of other members of *Flaviviridae*, such as bovine viral diarrhea virus [31] and hepatitis C virus [32-34], with very promising perspectives. Acridones affected the functionality of viral RNA in infections with both flaviviruses. The inhibition of hepatitis C virus enzymes like RNA helicase and RNA polymerase has been reported for a group of *N*-substituted acridone-4-carboxamides as well as the ability of these compounds for RNA intercalation, probably leading to the disruption of the interaction between the enzyme and the modified nucleic acid [34]. Then, the possibility of multiple targets to block viral RNA synthesis was suggested by the authors.

The antiviral activity of **3b** appears also related to inhibition of viral RNA synthesis as determined by real time PCR but the molecular mechanism has not been yet fully elucidated. The strong inhibition observed in DENV-2 infection may be partially restored by the simultaneous addition of guanosine together with the acridone, indicating a possible involvement of the cellular enzyme IMPDH as a potential target of this compound. This enzyme is an interesting host factor to be considered for wide spectrum antiviral chemotherapy [35]. In recent years, the targeting of host functions essential for virus replication has regained interest respect to the direct-acting antiviral agents. Besides the certain possibility to develop compounds active against a broad spectrum of viruses, the cell-based approach is expected to reduce the chances for emergence of drug-resistant viral variants. The disadvantage of this strategy is the higher potential for cell toxicity and undesirable effects, but it is not a drawback for acute diseases like dengue fever or dengue hemorrhagic fever that require short time of treatment. Furthermore, the toxicity of cellular protein-targeted compounds may be variable according to the compound design. The main challenge is to get an inhibitor with higher specificity to block viral infection in comparison to cell viability, as appears to be for acridone **3b** from data shown in Figure 1B. In particular, several IMPDH inhibitors with *in vitro* anti-DENV effectiveness in different mammalian cells have been reported, including well-characterized drugs such as RIB and mycophenolic acid [27,36] and also diverse classes of novel heterocyclic molecules [37-39]. Similarly, the cellular enzyme dihydroorotate dehydrogenase (DHODH), that is required for *de novo* pyrimidine biosynthesis, was also tested in recent studies as a novel and effective host-dependent target against DENV infection [40,41].

**Figure 6:**
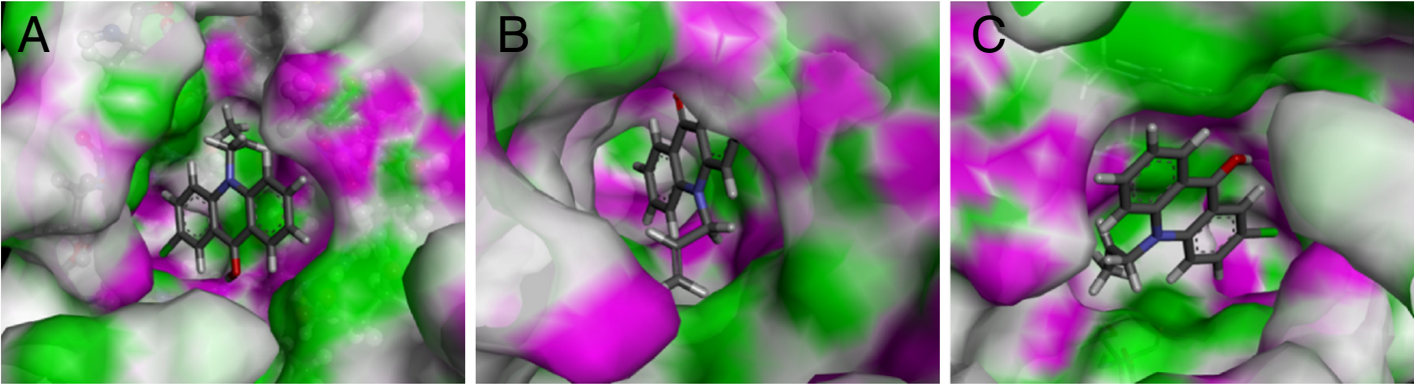
Prediction affinity about **3b**-IMPHD interaction **(A)** Binding of **3b** to IMPDH active site. **(B)** Binding of **3b** to RIB monophosphate inhibitory site. **(C)** Binding of **3b** to IMP activity site.

The molecular docking studies performed provide useful information about **3b**-IMPHD interaction. The binding orientation of **3b** to human IMPHD was found in order to predict the affinity and activity of the compound. As the docking results presented, **3b** showed the best-fit orientation when it was docked into active site, IMP and RIB monophosphate binding site of IMPHD (Figure 6).

A probable disadvantage to face for the *in vivo* efficacy of compounds targeting enzymes involved in purine or pyrimidine biosynthesis may be the presence of exogenous nucleotides in plasma that could be taken by the cell and reverse the inhibitory activity. However, this issue must be analyzed in each particular situation considering the ability of any compound to overcome this inconvenience. For example, it is crucial the concentration of exogenous purine or pyrimidine required for reversal of the inhibitory activity, a parameter that is not equivalent for all compounds affecting nucleotide biosyntesis. In addition it is also crucial the plasma binding activity of the inhibitor because it may be determinant for the plasma concentration of free compound to surpass the effect of external nucleotide supplementation [41]. Accordingly, the antiviral *in vivo* evaluation in animal models of IMPDH or DHODH inhibitors has reported variable results. Leflunomide and a thiophene-2-carboxylic acid derivative, two DHODH inhibitors, were effective against respiratory syncytial virus infection of cotton rats and cytomegalovirus infection of mice, respectively [42,43], whereas the DHODH inhibitor designated NITD-982 showed *in vitro* potency against DENV-2 but was inactive in the AG129 mouse model [41]. Recently, the chemical inhibition with mycophenolic acid or the depletion mediated by RNA interference of IMPDH was found effective to suppress *in vivo* DENV infection of *Aedes aegypti* mosquitoes, an interesting strategy for blockade in DENV transmission from vector to human and control of virus dissemination [44].

In our case, there is an incomplete recovery of DENV-2 infectivity by the presence of exogenous guanosine. Consequently, more than one target related to DENV RNA synthesis appeared responsible of the antiviral activity of **3b**, as above commented for other acridones and flaviviruses. A similar partial participation of IMPDH has also been reported for the antiviral mechanism of other *N*-substituted acridone derivative, the compound 10-allyl-6-chloro-4-methoxy-9(10*H*)-acridone, against JUNV [45]. Since this last drug is also inhibitory of DENV infection [21], the participation of cellular IMPDH in the antiviral activity of these heterocyclic compounds supports the wide spectrum of effectiveness of diverse acridones against unrelated viruses, like JUNV and DENV.

In conclusion, in the present study we demonstrated that the acridone derivative **3b** selectively inhibits the *in vitro* infection with the four DENV serotypes without a direct interaction with the host cell or the virion but interfering specifically with the intracellular virus multiplication. The cellular enzyme IMPDH appears to be partially involved in anti-DENV activity of acridone, but other unidentified target affecting viral RNA synthesis must also be active to accomplish for the virus yield inhibition produced by the drug, suggesting a dual mechanism of antiviral action. The *N-*allyl acridones are broad spectrum antiviral agents that represent a very promising tool for management of hemorrhagic fever-causing viruses lacking of specific chemotherapy. The inhibitory effectiveness of acridones against DENV and JUNV merits further testing of these compounds with other medically relevant RNA viruses.
